# Intestinal injury signaling pathway in sepsis

**DOI:** 10.3389/fimmu.2025.1620965

**Published:** 2025-06-27

**Authors:** Lin Liu, Qin Yue, Jinhao Chen, Hui Liu, Xinyi Zeng

**Affiliations:** Yangtzeu University Health Science Center, Jingzhou, Hubei, China

**Keywords:** sepsis, intestinal injury, NF-κB, signaling pathway, inflammatory response

## Abstract

Sepsis is a syndrome of inflammatory response syndrome (SIRS) triggered when the host is exposed to bacterial, viruses, and other infectious agents. The resulting inflammation compromises intestinal integrity, and this gut injury subsequently amplifies systemic inflammation, ultimately leading to multiple organ failure. This review synthesizes recent advances in sepsis-induced intestinal injury, focusing on four key aspects: pathogenesis, molecular mechanisms, crosstalk among relevant signaling pathways, and therapeutic strategies. Our analysis reveals extensive interactions between these signaling pathways, with most being critically dependent on nuclear factor-kappa B (NF-κB). We propose that the nuclear factor-κB signaling pathway serves as a central hub in the mechanistic network of septic gut injury. By delineating the interplay of signaling pathways in intestinal damage during sepsis. This work aims to provide novel therapeutic perspectives.

## 1 Introduction

Sepsis is a potentially fatal condition characterized by organ dysfunction stemming from an aberrant host response to infection, which may advance through systemic inflammatory cascades to septic shock and multiple organ dysfunction syndrome (MODS) (1). According to data from the Global Burden of Disease (GBD) study in 2017, the estimated mortality rate among global sepsis patients was 22.5%. Health impairments contributed to a sepsis incidence rate of 67.4%, of which sepsis complications caused by diarrheal diseases accounted for 12.2% (2). An extended systematic evaluation and meta-analysis further revealed that sepsis treated in hospitals occurred at an incidence of 189 per 100,000 person-years, with mortality exceeding 25%, while sepsis requiring ICU admission had an estimated incidence of 58 per 100,000 person-years (3). Consequently, sepsis, characterized by elevated morbidity and mortality, has emerged as a critical global health challenge (4).

Within the multiorgan dysfunction induced by sepsis, the gastrointestinal tract represents a primary target organ. Its barrier integrity, maintained by tight junction (TJ), between intestinal epithelial cells and secreted mucus, facilitates selective permeability while preventing translocation of luminal antigens, microorganisms, and their toxins into systemic circulation (5). When sepsis occurs, intestinal inflammatory factors exacerbate the systemic inflammatory response by activating intracellular signaling pathways in the intestinal cells. This leads to increased intestinal barrier permeability, bacterial translocation, and microbial dysregulation, which collectively impair intestinal immune function and promote the dissemination of organ dysfunction from the gut to systemic compartments, ultimately contributing to the development of MODS (6). In addition, studies have shown that nearly 50% of sepsis patients with intestinal injuries are treated in ICUs (7), and intestinal injury is strongly associated with septic shock and 28-day mortality (8). These findings underscore the vital role of the gut in alleviating sepsis and reducing sepsis-associated mortality.

To investigate the mechanisms of septic bowel injury, this review synthesized literature over the past decade on the pathogenesis of septic intestinal injury and identified potential involvement of multiple signaling pathways: the nuclear factor-κB (NF-κB) signaling pathway related to inflammatory response, the Toll-like receptors (TLRs) signaling pathway related to immune response, the Rho/Rock signaling pathway related to membrane motility, the phosphatidylinositol 3-kinase (PI3K)/protein kinase B (AKT) signaling pathway related to cellular autophagy, the NOD-like receptor pyrin domain-containing 3 (NLRP3)/caspase-1 signaling pathway related to cellular pyroptosis, the Wnt signaling pathway related to apoptosis and necroptosis, and the Nuclear factor erythroid 2-related factor 2 (Nrf2) signaling pathway that regulates oxidation. By dissecting the mechanistic roles of these pathways in septic bowel injury, this review aims to identify crosstalk between signaling networks and uncover novel therapeutic targets for septic intestinal injury.

## 2 Pathogenesis of sepsis in the intestine

Sepsis arises from the disruption of homeostasis between inflammation and immunosuppression, leading to uncontrolled systemic inflammation and immune system dysregulation (9). Intestinal injury in sepsis is characterized by intestinal barrier dysfunction, manifested as microbial dysbiosis, bacterial translocation increased intestinal permeability, and immune response suppression (10). These pathological processes form a positive feedback loop that exacerbates intestinal barrier injury and accelerates the dissemination of sepsis from the gut to multiple remote organs.

The intestinal epithelial barrier, composed of intestinal epithelial cells and their intercellular junctions, is a physical barrier between the outside world and the internal environment and plays a role in maintaining homeostasis in the body. This barrier comprises three major junction types: TJs, gap junctions (GJs), and adherens junctions (AJs). TJ complexes include key proteins such as occludin and zonula occludens-1 (ZO-1), among which ZO-1 dynamically regulates paracellular permeability by sealing intercellular spaces and controlling the selective transport of luminal substances (11).

The density and composition of the intestinal microbiota directly modulate intestinal barrier permeability and colonization resistance. Conversely, host immune status reciprocally shapes microbial community structure. Gut inflammation frequently arises from dysregulated immune responses to commensal microbiota, where loss of immunological tolerance triggers pathological inflammation (12). The intestinal immune response involves intestinal mononuclear phagocytes (MNPs), including tissue-resident macrophages (RMs), inflammatory macrophages (IMs), and dendritic cells (DCs). These cells not only clear intestinal pathogens, but also modulate the inflammatory response by regulating the release of inflammatory cytokines, including tumor necrosis factor-α (TNF-α), tumor necrosis factor-β (TNF-β), interleukin-1 (IL-1), interleukin-6 (IL-6), and interleukin-10 (IL-10) (13), which maintain intestinal epithelial barrier integrity, and promote immune cell maturation (14). The major bacterial groups in the gut include commensal bacteria, which constitute over half of the gut microbiota and primarily provide energy to the host (15). The intestinal commensal bacterium, Ackermannia, effectively reduces inflammation by enhancing the stabilization of the stabilization of the intestinal epithelial barrier and inhibiting the release of inflammatory cytokines (16).

**Figure 1:**
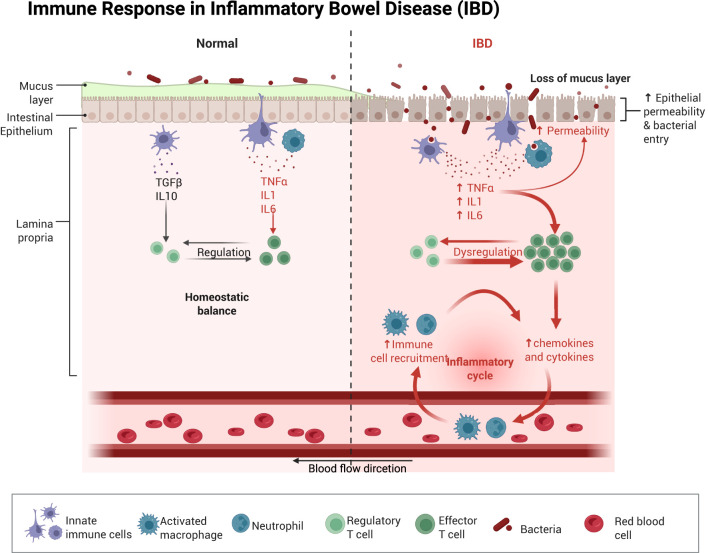
Schematic illustration of the immune response in the intestine after normal as well as impaired intestinal permeability. Once the intestinal barrier is damaged, intestinal dysbiosis occurs, and immune cells migrate from the bloodstream into intestinal tissues. This process promotes the upregulation of pro-inflammatory cytokines in the gut, leading to further exacerbation of intestinal barrier injury and subsequent induction of intestinal sepsis. Created with BioRender.com.

Gut microbiota dysbiosis can trigger hyperactivation of intestinal immune cells, leading to upregulated expression of inflammatory factors. This process enhances intestinal permeability, potentially causing translocation of bacteria, bacterial products, and other luminal contents (17). Disrupting the dynamic balance and composition of the intestinal flora allows bacteria and toxins from compromised intestines to translocate into the bloodstream, triggering multiorgan inflammation and infections. This process can lead to systemic inflammation, a key driver of sepsis pathogenesis (**Figure 1**) (13, 18). Meanwhile, sepsis progression induces excessive production of inflammatory factors in the intestine. This process enhances the expression of inducible nitric oxide synthase (iNOS) and stimulates nitric oxide (NO) synthesis (19). Furthermore, in the process of pathogen clearance, the recruitment and activation of neutrophil extracellular traps (NETs) coincides with endoplasmic reticulum, stress-induced production of reactive oxygen species (ROS), causing oxidative damage to the intestinal barrier (20).

## 3 Intestinal injury signaling pathway in sepsis

### 3.1 NF-κB signaling pathways

NF-κB, a transcription factor ubiquitously expressed in cells, orchestrates the transcriptional regulation of numerous inflammatory and immune-related genes (21). Elevated activation of NF-κB promotes robust expression of pro-inflammatory genes, thereby exacerbating the inflammatory cascade (22). Moreover, in response to oxidative stress, NF-κB serves as a central transcriptional regulator that profoundly influences both mitochondrial structure and energy metabolism (23).

**Figure 2:**
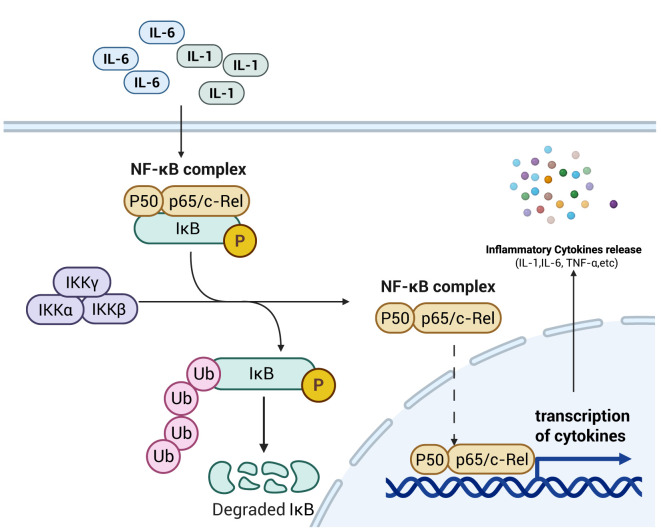
Schematic diagram of NF-κB signaling pathway mechanism. The increase in pro-inflammatory cytokines within the intestinal tract activates the NF-κB signaling pathway, promoting the transcription and translation of these cytokines. This process thereby enhances intestinal permeability, laying the foundation for the development of intestinal sepsis. Created with BioRender.com.

The NF-κB subfamily consists of five key members: NF-κB1 (also referred to as p50), NF-κB2 (also referred to as p52), RelA (often referred to as p65), c-Rel, and RelB (24). Under normal physiological conditions, the NF-κB inhibitory protein IκB binds to the p50/p65 heterodimer, forming a cytoplasmic complex that sequesters the transcription factor in the cytosol and blocks its nuclear translocation (25). As shown in **Figure 2**. Under normal physiological conditions, the NF-κB inhibitor IκB binds to the p50/p65 heterodimer, forming a cytoplasmic complex that sequesters the NF-κB transcription factor in the cytosol and inhibits its nuclear translocation (26). Concurrently, the activated NF-κB dimer translocates through the nuclear pore into the nucleus, where subsequent release of NF-κB enables its binding to κB response elements within promoter or enhancer regions of inflammatory target genes, thereby upregulating expression of these inflammatory factors (27), aggravating septic bowel injury. Furthermore, E2F1 (E2F transcription factor 1), a member of the E2F transcription factor family implicated in both cell cycle regulation and apoptosis, can interact with NF-κB to form the E2F1/NF-κB complex. This protein-protein interaction inhibits NF-κB p65 nuclear translocation by attenuating its phosphorylation status, thereby suppressing the NF-κB signaling pathway (28). Moreover, NF-κB modulates the intestinal epithelial barrier via transcriptional regulation of myocilin light chain kinase (MLCK) expression, thereby influencing intestinal epithelial permeability (29). At the post-transcriptional level, the NF-κB signaling pathway is also regulated by microRNAs (MiRNAs) (30). MiRNAs function as regulators through complementary base pairing with 3′-untranslated regions (3′-UTRs) of target mRNAs, resulting in mRNA degradation or translational repression. For instance, MiR-155 downregulates SIRT1 expression, promotes NF-κB dimerization, enhances NF-κB signaling activity, and exacerbates intestinal mucosal injury in septic rats (31). Conversely, MiR-199a-5p suppresses the expression of surfactant protein D (SP-D), a negative regulator of NF-κB, thereby triggering the NF-κB pathway and exacerbating intestinal barrier dysfunction in septic mice (32).

As a key signaling pathway governing the inflammatory response, the NF-κB pathway primarily modulates intestinal barrier permeability through two mechanisms: regulating the expression of pro-inflammatory cytokines in immune cells and controlling MLCK expression within intestinal epithelial cells. These actions collectively exert a critical influence on septic intestinal injury. Additionally, intracellular MiRNAs post-transcriptionally regulate key genes in the NF-κB signaling cascade, thereby modulating the quantity of NF-κB that ultimately translocates to the nucleus and binds to κB response elements.

### 3.2 Toll-like receptor signaling pathways

TLRs are a family of transmembrane proteins primarily responsible for mediating the body’s innate immune defense, functioning as the initial line of defense against disease-causing microbes. All family members share a conserved leucine-rich Toll/Interleukin-1 receptor (TIR) domain in their cytoplasmic region (33). TLRs detect damage-associated molecular patterns (DAMPs) and pathogen-associated molecular patterns (PAMPs), initiating signaling cascades that promote the synthesis of pro-inflammatory cytokines and chemokines (34). Additionally, TLRs can promote the induction of adaptive immune responses *in vivo (* 35).

**Figure 3:**
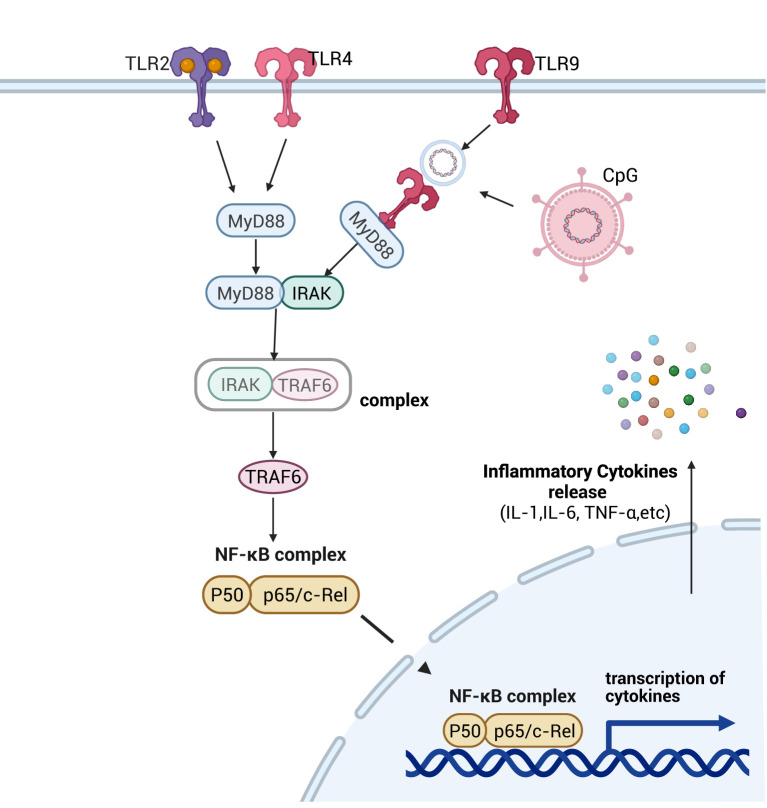
Schematic diagram of the signaling pathway mechanism of TLRs. After activation of different TLRs by pathogenic microorganisms in the intestine, with the help of MyD88-dependent as well as non-dependent pathways, the downstream NF-κB signaling pathway was activated, which promoted the expression of inflammatory factors and led to the impairment of intestinal permeability, which then contributed to the development of intestinal sepsis. Created with BioRender.com.

TLRs are primarily composed of three components: intracellular TIR domains, signal transduction cascades, and cross-regulation of signaling branches. Based on their subcellular localization, TLRs are categorized into two major groups: cell surface-localized TLRs (e.g., TLR1, TLR2, TLR4) that act at the plasma membrane, and intracellular TLRs (e.g., TLR7, TLR8, TLR9) that function within endosomal or cytoplasmic compartments (36). Among these, TLR2, TLR4 and TLR9 are the primary pattern recognition receptors (PRRs) in intestinal TLR signaling pathway, capable of recognizing a broad spectrum of microbial PAMPs and DAMPs (37). By contrast, TLR2 and TLR4 directly engage the downstream signaling adaptor protein myeloid differentiation primary response 88 (MyD88) to recruit and activate interleukin-1 receptor-associated kinase (IRAK) (38). In contrast, TLR9—a type I transmembrane protein—recognizes unmethylated CpG dinucleotides in bacterial or viral DNA within enterocytes, triggering its activation (39). Upon activation, MyD88 recruits IRAK via its death domain (DD), leading to rapid phosphorylation and activation of IRAK (40). Subsequently, activated IRAK dissociates from the complex, binds to and activates downstream tumor necrosis factor receptor-associated factor 6 (TRAF6), thereby prompting nuclear translocation of NF-κB dimers to regulate inflammatory factor expression (41), and exacerbate septic intestinal injury (**Figure 3**). This process constitutes a positive feedback loop, wherein NF-κB is further activated to upregulate inflammatory factor expression, establishing a signaling cascade that amplifies the inflammatory reaction (42). Additionally, cytoplasmic high mobility group protein box 1 protein (HMGB1) promotes inflammatory factor IL-33 expression by binding to the TLR4 receptor, thereby modulating the inflammatory response through this ligand-receptor interaction (43).

In the intestinal TLR signaling pathway, we observed that TLRs primarily recognize unmethylated DNA motifs (e.g., CpG dinucleotides) of intestinal pathogens and activate the NF-κB signaling cascade via MyD88-dependent signaling. This process elevates pro-inflammatory cytokine expression and exacerbates inflammatory cascades, culminating in the breakdown of intestinal immune homeostasis.

### 3.3 Rho/ROCK signaling pathways

Composed of small GTP-binding proteins, the Ras homolog (Rho) family is regulated by cell surface receptors, such as integrins, cytokine receptors and growth factor receptors. Rho family proteins are the founding members of the small guanosine triphosphatase (GTPases) superfamily, functioning as molecular switches with intrinsic GTPase activity. Canonical RhoGTPases include RhoA, CDC42 and RAC1 (44). These proteins regulate cell motility primarily by modulating cytoskeletal dynamics in response to extracellular signals (45). Downstream of RhoA, Rho-associated protein kinases (ROCKs) are serine/threonine kinases with two isoforms, ROCK1 and ROCK2, which mediate cytoskeletal reorganization and signaling events (46).

**Figure 4:**
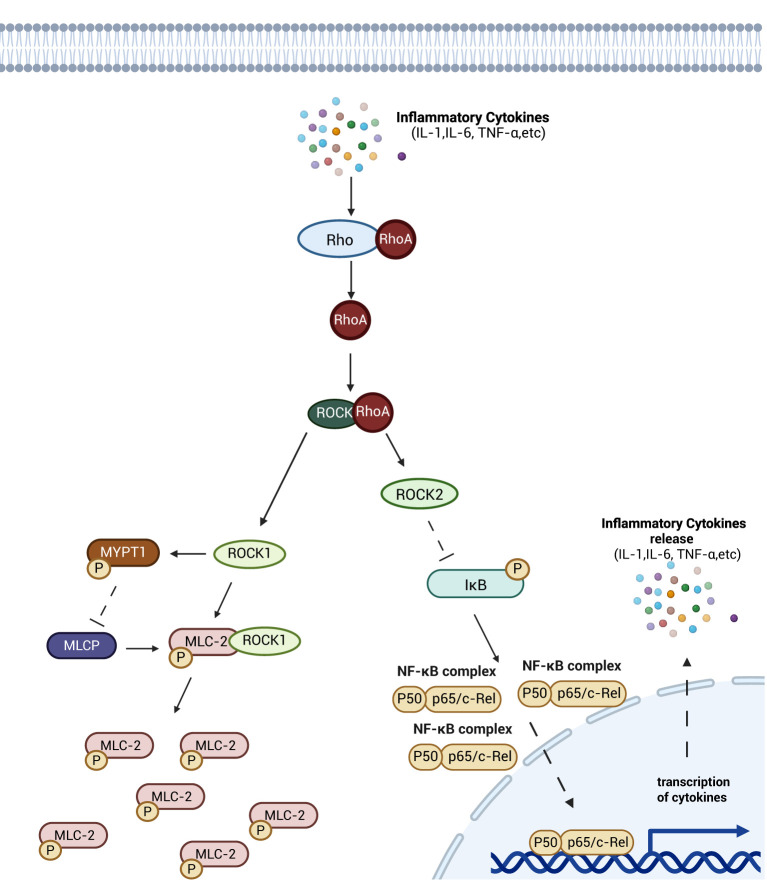
Schematic diagram of the mechanism of Rho/Rock signaling pathway. Rho gradually activates the downstream ROCK after activation of inflammatory factor. ROCKK1 inhibits the dephosphorylation of pMLC-2, and ROCK2 inhibits the activity of IκB, an inhibitor of NF-κB proteins, which leads to the entry of more NF-κB-active dimers into the nucleus, promoting the release of inflammatory factors, and aggravating the damage of intestinal barrier as well as contributing to intestinal sepsis through the two pathways. damage as well as contributing to the development of intestinal sepsis. Created with BioRender.com.

Rho, ROCK and myosin light chain (MLC) are the primary components constituting the Rho/ROCK signaling pathway, which regulates cytoskeletal dynamics in intestinal barrier cells (47). Upon activation by inflammatory stimuli, RhoA activates downstream ROCK1. ROCK1 directly phosphorylates myosin light chain II (MLC-2) and inhibits myosin light chain phosphatase (MLCP) activity by phosphorylating myosin phosphatase targeting subunit 1 (MYPT1), resulting in elevated intracellular levels of phosphorylated MLC-2 (pMLC-2). As a key regulator of intestinal epithelial junction integrity, increased pMLC-2 enhances permeability of the intestinal epithelial barrier, thereby inducing intestinal barrier damage (48, 49). In contrast, ROCK2—the other ROCK isoform—downregulates IκB expression, increasing cytoplasmic levels of activated NF-κB dimers and promoting their nuclear translocation (50), this mechanism amplifies immune and inflammatory responses in host cells by potentiating NF-κB signaling (51) (**Figure 4**).

The Rho/ROCK signaling pathway regulates intestinal epithelial cell-cell junctions by controlling MLC-II phosphorylation, thereby maintaining junctional integrity and directly modulating intestinal barrier permeability. Additionally, this pathway indirectly influences barrier permeability by regulating the abundance of intracellular activated NF-κB dimer.

### 3.4 PI3K/AKT signaling pathways

Phosphatidylinositol 3-kinase (PI3K) is a heterodimeric intracellular kinase composed of a catalytic subunit (p110) and a regulatory subunit (p85 or p84/p101), which is ubiquitously expressed in most mammalian cells and critically regulates cell cycle progression, survival, migration and growth (52). Protein kinase B (AKT), a multifunctional serine/threonine kinase, transduces signals by phosphorylating diverse downstream substrates. Among these, the mammalian target of rapamycin (mTOR) stands as a key downstream effector, governing cell proliferation, autophagy, and metabolic homeostasis (53).

**Figure 5:**
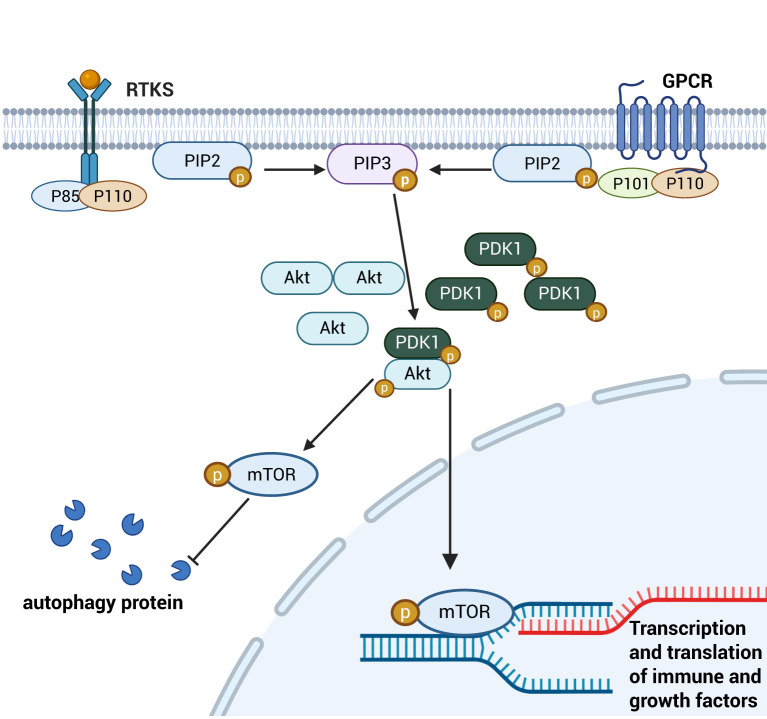
Schematic diagram of the PI3K/AKT signaling pathway. Cytosolic PI3K is activated and sequentially phosphorylates the key downstream target mTOR. This activation inhibits autophagy induction by phosphorylating cytoplasmic autophagy-related proteins. Concurrently, the PI3K/mTOR pathway promotes the nuclear translocation of downstream transcription factors, which drive the transcription and translation of immune modulators and growth factors. This dual mechanism enhances intestinal epithelial cell proliferation and mitigates inflammatory damage to the intestinal barrier. Created with BioRender.com.

The PI3K/AKT signaling pathway is a major autophagy-inhibitory signaling axis, critically regulating cell migration, proliferation, differentiation, apoptosis and inflammatory factors expression (54). This pathway serves as a key mechanism for intestinal mucosal repair by modulating TJ protein synthesis, enhancing intestinal barrier function, and promoting intestinal epithelial cell proliferation, ultimately maintaining the integrity of the Gut barrier (55). Stimulated by signaling molecules, receptor tyrosine kinases (RTKs) and G protein-coupled receptors (GPCRs) on the surface of intestinal epithelial cells activate a downstream PI3K heterodimer consisting of a catalytic subunit, p110 and a regulatory subunit, p85. This activation catalyses the conversion of phosphatidylinositol 3,4-bisphosphate (PIP2) to phosphatidylinositol 3,4,5-trisphosphate (PIP3) on the irepairaflet of the cell membrane. SubsequAktly, PIP3 recruits Akt to the membrane, which is activated by phosphatidylinositol-dependent kinase 1 (PDK-1)-mactivated NFhorylation of Akt. Activated Akt migrates into cytoplasmic or nuclear compartments, executing phosphorylation of downstream effector molecules, including mTOR proteins, to regulate processes such as cell proliferation, survival and autophagy. Phosphorylated mTOR regulates proliferation and autophagy of intestinal immune cells and epithelial cells by inhibiting the expression of autophagy-related proteins. Through cytoplasmic signaling, it promotes immune responses by enhancing the translation of growth factors and pro-inflammatory cytokines, while also modulating intestinal barrier permeability via tight junction protein regulation. Although mTOR primarily functions in the cytoplasm, its downstream targets can translocate to the nucleus to influence transcription of genes involved in inflammation and epithelial repair (56–58)(See **Figure 5**). Furthermore, AKt activation indirectly regulates intestinal inflammatory responses by modulating the nuclear translocation of activated NF-κB dimers and suppressing NF-κB signaling activity (59, 60). Moreover, the PI3K/Akt pathway alleviates septic injury to the intestinal barrier by regulating cytoskeletal dynamics via Rho/ROCK signaling, thereby maintaining epithelial junction integrity (61).

As a major autophagy-inhibitory signaling pathway, PI3K/AKT regulates proliferation and autophagy of intestinal immune cells and epithelial cells by modulating the phosphorylation status of its downstream target mTOR. Activated mTOR suppresses autophagy-related protein expression, thereby controlling intestinal inflammatory factor production and modulating intestinal barrier permeability. Additionally, the PI3K/AKT pathway influences NF-κB signaling by promoting IκB phosphorylation, which prevents NF-κB dimer nuclear translocation and suppresses inflammatory responses. Collectively, these mechanisms enable PI3K/AKT to alleviate sepsis-induced intestinal injury by coordinating autophagic, inflammatory, and barrier regulatory networks.

### 3.5 NLRP3/caspase-1 signaling pathways

The NLRP3 inflammasome is a multiprotein complex acting as a cellular sensor that recognizes diverse microbial-associated molecular patterns (MAMPs), endogenous danger signals, and exogenous stimuli. It consists of three core components: the NLRP3 sensor (recognition component), the adaptor protein apoptosis-associated speck-like protein containing a CARD (ASC), and the effector pro-caspase-1 (62). Upon activation, the NLRP3 inflammasome orchestrates inflammatory immune response by facilitating the activation of downstream caspase-1, which drives pyroptotic cell death and promotes the maturation and secretion of pro-inflammatory cytokines IL-1β (pro-IL-1β) and IL-18 (pro-IL-18). This cascade ultimately exacerbates pyroptosis in macrophages and amplifies systemic inflammatory responses (63).

The triggering of NLRP3 inflammasome requires dual signaling. Firstly, upon activation of the NF-κB signaling pathway, NF-κB translocation to the nucleus promotes the transcription of the NLRP3 inflammasome gene, which upregulates the expression of NLRP3 inflammasome and prepares the cell for subsequent activation; Secondly, when mitochondrial dysfunction, lysosomal rupture, ROS production and disturbances in ion flow occur in cells, they combine to promote the oligomerisation of NLRP3, the release of the adaptor protein ASC, and their binding to pro-caspase-1 to form a multiprotein complex (64). Upon activation, NLRP3 inflammasome interacts with ASC, the Caspase Recruitment Domain (CARD) of ASC then engages the CARD domain of pro-Caspase-1, facilitating its oligomerization and autocleavage into active caspase-1 (65). Activated caspase-1 not only converts inactive precursors of IL-1β and IL-18 into mature, active forms, but also creates perforations in the cell membranes by cutting the GSDMD, leading to sustained cellular expansion until rupture, releasing intracellular pro-inflammatory factors, and exacerbated inflammatory responses (66). Therefore, inhibiting NLRP3 inflammasome activation to reduce downstream caspase-1 proteolytic activity—thereby attenuating pyroptotic cell death and suppressing the maturation and secretion of pro-inflammatory cytokines—represents a validated strategy to alleviate inflammatory injury. Upregulation of the vitamin D receptor (VDR) expression inhibits NLRP3 inflammasome activation by suppressing upstream NF-κB signaling, thereby reducing caspase-1-mediated maturation and secretion of IL-1β/IL-18 and attenuating pyroptotic death of intestinal macrophages. This dual inhibition of inflammation and pyroptosis preserves the intestinal mucosal barrier by minimizing epithelial damage and immune cell-driven tissue injury (67). Meanwhile, it can also activate the Akt/mTOR pathway to promote cellular autophagy and mediate the inactivation of NLRP3 inflammasome, thereby reducing the inflammatory response and alleviating intestinal tissue damage in septic rats (68). In addition, inhibition of NF-κB phosphorylation, suppresses the priming phase of NLRP3 inflammasome activation, thereby reducing transcriptional upregulation of NLRP3, pro-IL-1β, and pro-IL-18. This intervention attenuates inflammatory cytokine maturation and suppresses pyroptotic cell death in intestinal epithelia and immune cells (69, 70).

The activation of NLRP3 as a downstream target of the NF-κB signaling pathway requires not only the activation of NF-κB, but also triggers the oligomerisation of NLRP3 and its assembly with ASC and pro-caspase-1. The activated NLRP3 inflammasome will cause rapid activation of pro-inflammatory factors in the intestinal tract, and continuously cause cellular autophagy, which step by step exacerbates the inflammatory response, leading to more and more severe intestinal injury.

### 3.6 Wnt signaling pathways

The Wnt pathway constitutes a phylogenetically preserved molecular framework governing critical events including: embryonic patterning, lineage-specific differentiation, and homeostatic tissue renewal (71). Wnt ligand-bound seven-pass transmembrane receptor complex consists mainly of the Frizzled (Fzd) family proteins and its co-receptor complexes, including low-density lipoprotein receptor-related proteins (LRP5/6) (72). Wnt proteins are activated through interactions with LRP5/6 and Fzd family receptors, leading to signaling from the extracellular to the intracellular (73).

**Figure 6:**
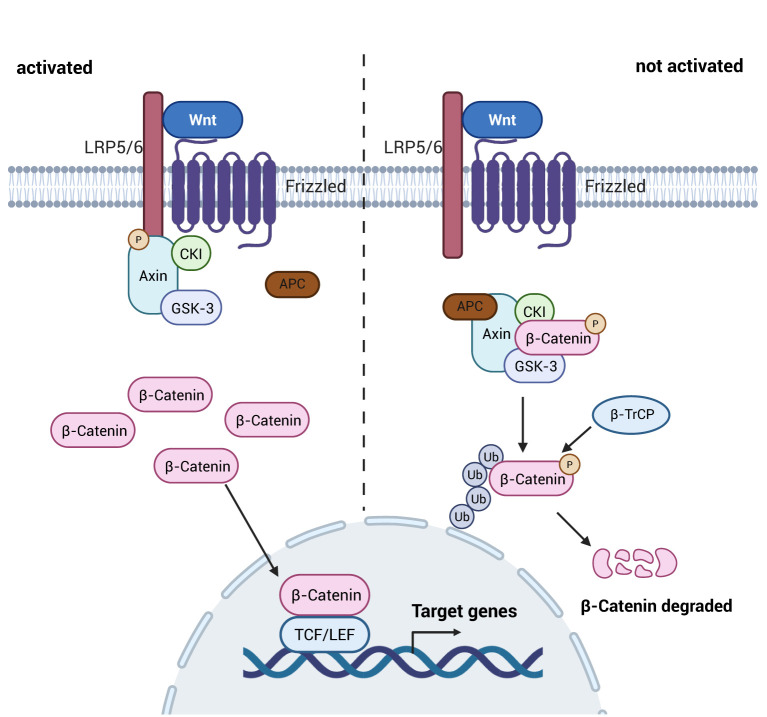
Schematic diagram of the mechanism of the Wnt signaling pathway. Upon activation of the Wnt signaling pathway, the multiprotein complex dissociates, allowing unubiquitized and degraded β-catenin to enter the nucleus, resulting in an increase in the expression of anti-inflammatory factors and mitigating the damage caused by inflammatory factors to the intestine; whereas, when it is not activated, the multiprotein complex ubiquitinates as well as degrades the β-catenin after it has been phosphorylated, and it fails to play its role as an anti-inflammatory agen. Created with BioRender.com.

The Wnt/β-catenin signaling pathway is the most common of the Wnt signaling pathways and consists mainly of proteins of the Wnt family, including: Wnt 3a, β-catenin, Adenomatous Polyposis Coli (APC), casein kinase 1 (CK1), glycogen synthase kinase 3β (GSK-3β), and Axin (74). When the intracellular Wnt signaling pathway is not activated, β-catenin, APC, GSK3, CK1 and Axin in the cytoplasm together form a multiprotein degradation complex, which phosphorylates part of β-catenin in the cytoplasma, which is then ubiquitylated and degraded by recognition of the E3 ubiquitin ligase substrate (β-TrCP), and at the same time, causes β-TrCP is highly expressed, leading to increased degradation of IκB-α and enhanced trans-activation of NF-κB (75, 76), thereby promoting intestinal inflammation, i.e., there is a negative regulatory interaction between the Wnt pathway and the NF-κB signaling pathway. When the upstream Wnt signaling pathway is in the activated state, the cytoplasmic tail of LRP5/6 recruits Axin into the complex via phosphorylation by Fzd/Dvl. This process leads to the dissociation of the complex from the cytoplasm, where it accumulates due to the inability of β-catenin to be degraded, prompting the entry of β-catenin into the nucleus, where they bind and are expressed by the T-cell factor (TCF) and lymphoid enhancer factor binding factor (LEF) families, which induces the expression of anti-inflammatory factors (73), as shown in **Figure 6**. Additionally, activation of Wnt signaling promotes the proliferation of intestinal crypt stem cells, which retains self-propagating ability with broad differentiation plasticity. These stem cells rapidly replace damaged intestinal epithelial cells, thereby maintaining intestinal homeostasis, restoring epithelial barrier integrity, and facilitating repair of intestinal injury following inflammatory or ischemic insults (77). Canonical Wnt/β-catenin signaling activity exhibits a well-defined gradient along the intestinal crypt-villus axis, peaking at the crypt base and gradually diminishing toward the villus tip. This spatial organization is critical for maintaining intestinal epithelial homeostasis (78).

The classical pathway of Wnt signaling pathway is an important pathway in the intestine to regulate the immune response as well as intestinal permeability. When the pathway is activated the high expression of β-catenin can promote the expression of anti-inflammatory factors in the immune cells, and at the same time inhibit the release of pro-inflammatory factors from the NF-κB signaling pathway, so as to maintain the homeostasis of the immune response as well as intestinal permeability in the intestine. In addition, the Wnt signaling pathway also affects cytoskeletal components and influences the intestinal barrier (61).

### 3.7 Red lineage nuclear transcription factor 2-related factor 2 signaling pathways

Nrf2 is a transcription factor involved in oxidative stress and antioxidant damage. Activation of Nrf2 helps cells to fight against damage caused by inflammatory responses. In addition, it is involved in cellular metabolism (79).

**Figure 7:**
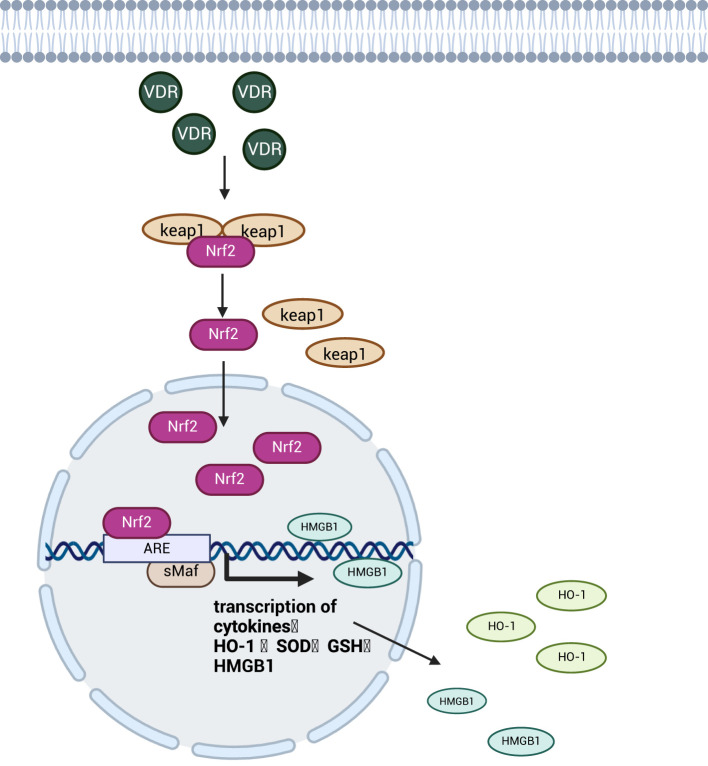
Schematic diagram of the mechanism of the Nrf2 signaling pathway. The Nrf2 transcription factor enters the nucleus after activation by the VDR and promotes the expression of antioxidant enzymes upon binding to the transcriptional progenitor ARE, which attenuates oxidative damage to intestinal cells and protects the integrity of the intestinal barrier. Created with BioRender.com.

The transcription factor Nrf2 and its negative regulator kelch-like ECH-associated protein 1 (Keap1) together constitute the Nrf2 signaling pathway. Keap1 acts as a substrate adaptor for E3 ubiquitin ligase complex, maintains a low intracellular concentration of Nrf2 by targeting and binding to the transcription factor Nrf2, ubiquitinating it and tightly regulating its activity through proteasome-dependent degradation (80). Under conditions of severe inflammation and oxidative stress, vitamin D-related receptor (VDR) activation can enhance Nrf2 signaling through transcriptional or post-translational mechanisms. This promotes Nrf2 dissociation from its cytoplasmic repressor, Keap1, allowing Nrf2 to translocate to the nucleus and accumulate (81). Subsequently Nrf2 binds to small Maf proteins (sMaf) to form a heterodimer that activates the antioxidant response element (ARE) and promotes the transcription and expression of downstream antioxidant enzymes genes such as Heme Oxygenase-1 (HO-1), HMGB1, superoxide dismutase (SOD), and glutathione (GSH) (82), reduces oxidative stress levels and mitigates intestinal mucosal damage (83), as shown in **Figure 7**. In addition, among many antioxidant enzymes regulated by Nrf2, HO-1 protects intestinal epithelial cells by catalyzing the degradation of heme into three bioactive molecules: bilirubin, carbon monoxide (CO) and ferrous iron (Fe^2+^) (84). A study found that Nrf2 regulates HMGB1 to ameliorate intestinal injury in septic mice (85). This may be related to the fact that HMGB1 in the nucleus facilitates the repair of damaged and deformed DNA sequences, thereby inhibiting cellular oxidative damage (86). While Nrf2 exhibits a significant antagonistic effect on the NF-κB signaling pathway by both inhibiting oxidative stress to suppress NF-κB activation through promoting the expression of SOD and preventing the ubiquitination and proteasomal degradation of IκB to inhibit NF-κB nuclear translocation (87). Ultimately plays a role in attenuating intestinal damage in sepsis.

The Nrf2 signaling pathway reduces sepsis-induced intestinal inflammatory response and oxidative damage to epithelial cells by promoting anti-inflammatory and antioxidant enzyme expression while antagonizing NF-κB signaling to suppress pro-inflammatory factors and protect intestinal barrier integrity.

## 4 Interactions between signaling pathways

We have inductively found that there are interactions between the different signaling pathways affecting intestinal injury in sepsis. For example, Rho/ROCK, PI3K/AKT and Wnt signaling pathways all regulate the intestinal epithelial cytoskeleton, a key structure of the intestinal barrier, but there are differences in the effects of the three on the gut. The Rho/ROCK signaling pathway, when stimulated by inflammatory factors, disrupts the integrity of the intestinal epithelial cytoskeleton and impairs the intestinal barrier; the PI3K/AKT signaling pathway accelerates the synthesis of the intestinal epithelial cytoskeleton by promoting the assembly of TJ proteins, thereby enhancing the intestinal barrier function; the Wnt signaling pathway contributes to the reorganization of the damaged intestinal epithelial cell skeleton upon activation. In addition, HMGB1 has a dual role, amplifying the inflammatory response and exacerbating intestinal injury in the acute phase of intestinal injury, and participating in tissue remodeling and repairing the damage in the late phase of intestinal injury signaling pathway and Nrf2 signaling pathway produce different effects by regulating HMGB1 in the cytoplasm and nucleus, respectively (85). TLR4 in the TLRs signaling pathway binds to HMGB1 in the cytoplasm and promotes the expression of the inflammatory factor IL-33 to produce inflammatory responses, whereas the Nrf2 signaling pathway regulates HMGB1 in the nucleus, repairs the damaged and deformed DNA sequences, and attenuates the oxidative damage and protects intestinal epithelial cells.

**Figure 8:**
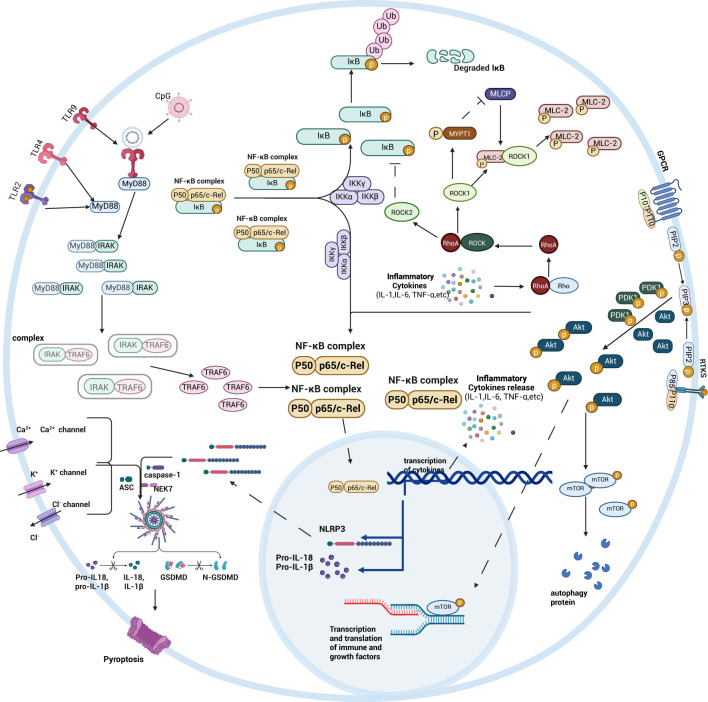
Schematic diagram summarizing the mechanisms of intestinal signaling pathways. By bringing together NF-κB, TLRs, Rho/ROCK, PI3K/AKT, and NLRP3/caspase-1 related signaling pathways, the role of the NF-κB signaling pathway in different signaling pathways is more clearly seen. Created with BioRender.com.

Interestingly, different signaling pathways affecting intestinal injury in sepsis are all inextricably linked to the NF-κB signaling pathway. In the PI3K/AKT signaling pathway, the activation of Akt also promotes the activation of the downstream NF-κB p65, which in turn activates the NF-κB signaling pathway, promotes the expression of inflammatory factors, and exacerbates septic bowel injury. When the Wnt signaling pathway is not activated, degradation of β-catenin in the cytoplasm results in high expression of β-TrCP, which in turn leads to degradation of IκB-α and activates the NF-κB signaling pathway, promoting intestinal inflammation. ROCK2 in the Rho/ROCK signaling pathway can regulate IκB expression, increase the number of active NF-κB dimers in the nucleus, and enhance intestinal barrier permeability, ultimately damaging the intestinal tract. The NLRP3/caspase-1 signaling pathway also increases the number of NF-κB active heterodimers. Differently, the Nrf2 signaling pathway ameliorates septic bowel injury by inhibiting the production of NF-κB active heterodimers by preventing the ubiquitination and degradation of the IκB proteasome(**Figure 8**).

## 5 Methods of treatment

The current treatments of intestinal injury in sepsis include antimicrobial therapy, nutritional approach therapy, probiotic therapy and drug therapy.

Antimicrobial therapy typically involves administering antibiotics to eliminate extracellular bacteria in the bloodstream or tissues, thereby reducing systemic inflammation. However, its limitations are significant: antibiotics primarily act on the bacteria in the extracellular space and are often ineffective against intracellular bacteria. Additionally, overuse or misuse of antibiotics drives the development of bacterial resistance, diminishing treatment efficacy and potentially leading to therapeutic failure (88). Nutritional therapy addresses disease-induced nutritional depletion by delivering adequate nutrients to the gastrointestinal tract, while also regulating intestinal, immune function to maintain the intestinal mucosal barrier and promote tissue repair (89). Probiotic therapy mitigates sepsis-induced, intestinal injury by preserving intestinal microecological stability, enhancing systemic antioxidant capacity, and modulating intestinal immunity (90). However, the optimal dosage and long-term safety of probiotics remain subjects of ongoing debate (91). For medication, different drugs are injected to help the body reduce the damage caused by sepsis, including anti-hyperglycaemic drugs (92), catecholamines, statins (88), Traditional Chinese medicine (93), and so on.

## 6 Discussion and prospects

Sepsis is an organ dysfunction syndrome triggered by infection, and the intestine is the most vulnerable target organ affected. We have found that the exacerbation of the intestinal inflammatory cascade represents its core pathological feature. This study elucidates that the NF-κB signaling pathway forms a complex interaction network with other signaling pathways to regulate intestinal inflammation. Different pathways can bidirectionally modulate inflammation intensity by either synergistically activating or antagonistically inhibiting the same effector molecules. Furthermore, distinct signaling pathways can also converge to induce the same pathological state (such as intestinal barrier impairment) by regulating different upstream and downstream targets. Targeted exploration of these interaction nodes for combined interventions holds promise for achieving multi-target synergistic effects in treating sepsis-induced intestinal injury. This strategy may overcome the therapeutic bottlenecks associated with inhibiting single pathways, offering novel approaches to address key clinical challenges in sepsis treatment, with the goal of reducing sepsis mortality. It holds significant clinical translational value.

In recent years, research on sepsis-induced intestinal injury has proliferated, yet investigations into its mechanisms largely remain confined to superficial analyses of isolated signaling pathways. The prevailing research paradigm suffers from dual limitations: Firstly, it oversimplifies signaling pathway research. Within complex networks encompassing tens of thousands of genes, investigators typically focus only on a minority of genes exhibiting significant expression differences. Secondly, it neglects interaction mechanism studies. This approach fails to discern whether highly expressed genes might result from synergistic regulation by multiple pathways, and similarly risks underestimating genes whose expression may be suppressed by negative crosstalk inhibition, thus appearing insignificant. These limitations severely constrain the in-depth analysis of the core mechanisms underlying sepsis-induced intestinal injury. We hypothesize that breakthrough therapeutic targets may reside within these pathway interaction nodes, potentially among genes showing non-significant differential expression, and are urgently awaiting systematic discovery through integrated multi-omics strategies.
